# Roles of circular RNAs in immune regulation and autoimmune diseases

**DOI:** 10.1038/s41419-019-1744-5

**Published:** 2019-06-26

**Authors:** Zheng Zhou, Bao Sun, Shiqiong Huang, Lingling Zhao

**Affiliations:** 1 Department of Chinese Medicine, The First Affiliated Hospital of Zhengzhou University, Zhengzhou, 450000 China; 2 Department of Clinical Pharmacology, Xiangya Hospital, Central South University, Changsha, 410000 China; 3 Hunan Key Laboratory of Pharmacogenetics, Institute of Clinical Pharmacology, Central South University, Changsha, 410000 China

**Keywords:** Immunopathogenesis, Autoimmunity

## Abstract

Circular RNAs (circRNAs), as a novel class of endogenously expressed non-coding RNAs (ncRNAs), have a high stability and often present tissue-specific expression and evolutionary conservation. Emerging evidence has suggested that circRNAs play an essential role in complex human pathologies. Notably, circRNAs, important gene modulators in the immune system, are strongly associated with the occurrence and development of autoimmune diseases. Here, we focus on the roles of circRNAs in immune cells and immune regulation, highlighting their potential as biomarkers and biological functions in autoimmune diseases, such as systemic lupus erythematosus (SLE), rheumatoid arthritis (RA), multiple sclerosis (MS), primary biliary cholangitis (PBC), and psoriasis, aiming at providing new insights into the diagnosis and therapy of these diseases.

## Facts

- CircRNAs are related to various biological processes in immune cells, as well as immune regulation under multifarious physiological and pathological conditions.
- CircRNAs serve as potential biomarkers for the diagnosis and severity of certain autoimmune diseases, such as systemic lupus erythematosus (SLE), rheumatoid arthritis (RA), multiple sclerosis (MS), primary biliary cholangitis (PBC).
- CircRNAs contribute to the development of autoimmune diseases by acting as miRNA sponges to regulate many biological processes, including DNA methylation, immune response, and inflammatory response.
- Certain circRNAs, such as cia-cGAS and dsRNA-containing circRNAs, may act as potential targets for the treatment of autoimmune diseases.

## Open questions

- What is the molecular mechanism by which circRNAs trigger autoimmune diseases?
- Are circRNAs effective and universal biomarkers for the diagnosis and severity of autoimmune diseases?
- How autoimmune diseases are linked to circRNAs' biogenesis, cytoplasmic accumulation and even post-transcriptional modifications?
- Is there potential for practical clinical applications based on findings concerning certain circRNAs?

## Introduction

Normally, immune cells have receptors that can distinguish between self (ie, healthy native structures) and nonself or deviant self (ie, pathogens or tumor antigens), enabling these cells to discover pathogens or malignantly transformed cells. At the same time, precise regulation of certain immune-related genes is essential to an organism’s ability to generate strong immunity to pathogens while limiting autoimmunity to self-antigens. Once immunodeficiency or immune dysregulation, people may suffer from immune system diseases, chronic infections, and even cancer. Typically, autoimmune diseases are a type of complex multifactorial diseases with characteristics of the presence of autoreactive immune cells and specific autoantibodies. According to statistics, there are >100 human diseases in the world that are known as autoimmune or chronic inflammatory, which are believed to affect 5–10% of individuals^1^.

Generally, circular RNAs (circRNAs) are widely studied non-coding RNA (ncRNA). Although RNA molecules are traditionally considered to be passive carriers of genetic information from DNA sequences to protein synthesis, lots of research has revealed that ncRNAs are critical participators in the process of gene expression^2,3^. To date, accumulated evidence has shown that circRNAs play an important role in various physiological and pathological processes, such as cancer^4,5^, cardiovascular diseases^6^, and neuronal diseases^7^. Intriguingly, circRNAs serve important functions in antiviral immunity^8^. Furthermore, circRNAs are aberrantly expressed in patients with systemic lupus erythematosus (SLE), some of which may serve as new non-invasive biomarkers for this autoimmune disease^9^. Therefore, an in-depth study of circRNAs will not only increase our understanding of the molecular mechanisms that underlie autoimmune diseases, but also provide future potential treatment of these diseases. In this review, we emphasize the potential roles of circRNAs in certain autoimmune diseases, including SLE, rheumatoid arthritis (RA), multiple sclerosis (MS), primary biliary cholangitis (PBC), and psoriasis.

## Biogenesis and functions of circRNAs

Unlike the characteristics of linear RNA molecules, circRNA has a special structure that is a covalently closed loop without 5' end caps and 3' Poly (A) tails^10,11^. This RNA species was first identified in RNA viruses in 1976^12^ and subsequently discovered in the cytoplasm of eukaryotic cells^13^ and yeast mitochondria^14^. With the development of high-throughput sequencing technology and microarray technique, plenty of circRNAs have been successfully discovered in various organisms in nature. In most cases, circRNAs are produced by “back-splicing” events of the precursor messenger RNAs (pre-mRNAs), in which a downstream 5' splice donor is linked to an upstream 3' splice acceptor via a 3' → 5' phosphodiester bond^15,16^. According to their components, circRNAs are mainly divided into three types: exonic circular RNAs (ecircRNAs)^17^, intronic circular RNAs (ciRNAs)^18^, and exon–intron circular RNAs (EIciRNAs)^19^, among which ecircRNAs occupy the vast majority.

**Fig. 1:**
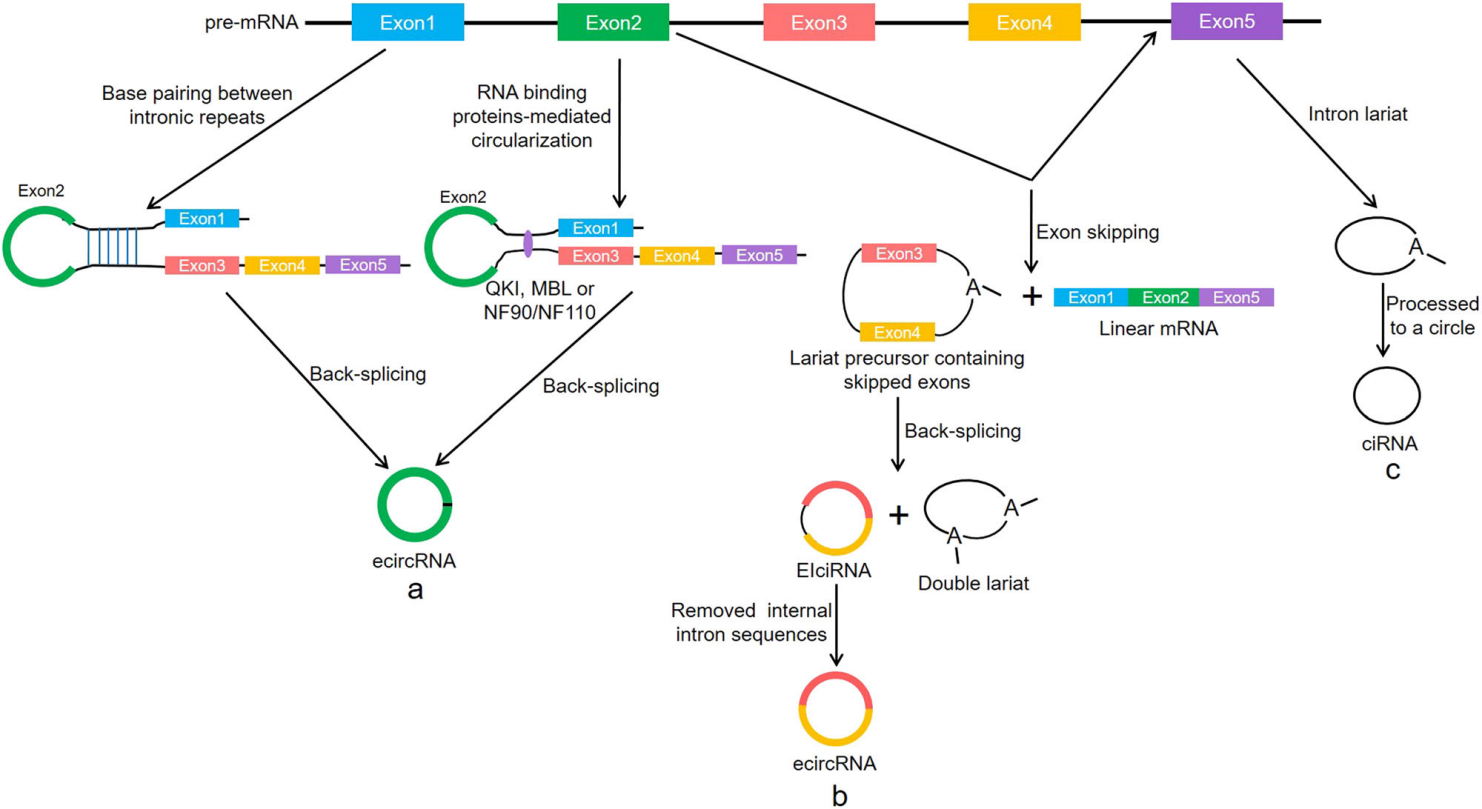
Formation of circRNAs by pre-mRNA back-splicing events. **a** The intron complementary repeat sequences or RNA-binding proteins such as QKI, MBL, and NF90/NF110 promote the back-splicing. **b** A lariat containing one or more skipped exons is re-spliced to generate a circRNA and a double lariat, and the circRNA may be an EIciRNAs or an ecircRNA. **c** CiRNA is generated from the intron lariat

Previous studies have found that RNA polymerase II (Pol II) elongation rate is associated with the efficiency and results of splicing^20,21^. The fast Pol II elongation may facilitate reverse complementary sequences across long flanking introns to pair up for back-splicing, thereby promoting circRNA formation^22^. Several possible circRNA biogenetic pathways, including “complementary sequence-mediated circularization”, “lariat-driven circularization” and “RNA-binding proteins-mediated circularization”, have been proposed. Liang et al.^23^ found that certain introns containing both splice sites and flanking inverted complementary repeats, such as Alu elements, were necessary for the circularization of the intervening exons in cells. In this process, the intronic repeat sequences must be base-paired with each other to bring the splice sites close together, thereby facilitating back-splicing. Notably, when a pre-mRNA has multiple intronic repeat sequences, the competitive pairing between the repeat sequences result in alternative circularization, thus affecting the splicing outcomes^24^. For example, this alternative circularization can cause a single gene to form multiple different circRNA transcripts^25^. Another form of circRNA generation is associated with exon skipping, in which a lariat precursor containing one or more skipped exons is first generated^26,27^. Then, the lariat removes its internal intron sequences to generate a mature circRNA and a double lariat. In some situations, the intervening introns in the encircled exons are not removed, which yields the so-called EIciRNA^16^. In addition, some RNA-binding proteins including the muscleblind, nuclear factor 90/nuclear factor 110 (NF90/NF110) and alternative splicing factor Quaking (QKI) were reported to promote back-splicing events by increasing the interaction between upstream and downstream introns^28–30^. CiRNAs are produced by intron lariats that fail to be degraded and debranched, and they do not contain linear 3' tails^18^. To some extent, these models explain the molecular mechanisms of circRNA biosynthesis (Fig. 1).

Usually, circRNAs work as molecular sponges for microRNAs (miRNAs), thereby regulating miRNA target gene expression^31,32^. Hansen et al.^31^ first found that a highly expressed circRNA (ciRS-7) could be used as a miR-7 sponge in human and mouse brains. This circRNA contained >70 conserved binding sites for miR-7, and it was strongly inhibited miR-7 activity by binding to miR-7, resulting in elevated levels of miR-7 targets. Subsequently, increasing evidence showed that various circRNAs could adsorb miRNAs, thus participating in many physiological and pathological processes^33,34^. For example, Hsa_circ_0009361 could regulate the expression of adenomatous polyposis coli 2 by binding to miR-582, thereby inhibiting the progression of colorectal cancer^35^. Furthermore, a class of EIciRNAs localized in the nucleus promoted the transcription of their parental genes in cis through interacting with U1 snRNP, indicating that these circRNAs could regulate gene expression via specific RNA–RNA interplay between U1 snRNA and EIciRNAs^19^. Conn et al.^36^ demonstrated that a circRNA derived from the exon 6 of SEPALLATA3 gene bound to its cognate DNA locus to form an R-loop and thus regulated the splicing of its cognate mRNA. Notably, the circular form of long intergenic non-protein-coding RNA p53-induced transcript could encode an 87 amino-acid regulatory peptide, which bound to polymerase associated factor complex (PAF1c) and inhibited the transcriptional elongation in glioblastoma^37^. These findings show that there may be more biological functions of circRNA than previously predicted.

## circRNAs in immunity

### circRNAs in immune cells

Indeed, some studies have demonstrated the diverse biological functions of circRNAs in immune cells. Hematopoietic stem cells (HSCs) can differentiate into a variety of progenitor cells, which subsequently generate all kinds of specialized blood cells, such as red blood cells, megakaryocytes, myeloid cells, and lymphocytes^38^. Nicolet et al.^39^ found that circRNA showed cell-specific expression in human hematopoietic progenitors as well as differentiated lymphoid and myeloid cells. For example, during hematopoietic differentiation, the expression levels of circRNA of lymphocytes were the highest, and the high levels were reflected in abundance rather than variety. Moreover, circ-FNDC3B showed the highest expression level in natural killer cells, while circ-ELK4, circ-MYBL1, and circ-SLFN12L showed the highest expression in T cells and natural killer cells. Macrophages are an essential part of innate immunity and can be induced to diverse phenotypes under different external stimuli^40^. A recent study explored the expression of circRNAs in macrophages under two different polarization conditions (M1 macrophages induced by interferon-γ (IFN-γ) and lipopolysaccharide (LPS), and M2 macrophages induced by interleukin-4 (IL-4))^41^. The results showed that 189 circRNAs were differentially expressed in the M1 compared with the M2 macrophages. To further elucidate the implication of the differentially expressed circRNAs, the researchers also predicted the miRNAs that interacted with them. For the overexpressed circRNA-010231 in M1, the five miRNA response elements with good scores were miR-1964-5p, miR-19b-2-5p, miR‑141-5p, miR-6950-5p, and miR-145a-5p, respectively. These findings provide new ideas for the roles of circRNAs in the polarization of macrophages. Recently, Agirre et al.^42^ found that 1356 new identified circRNAs were expressed in human humoral immune response, as well as plasma cells (tonsillar plasma cells and bone marrow plasma cells) had the highest average expression levels. The expression of these circRNAs was significantly negatively correlated with the levels of some RNA-binding proteins including adenosine deaminase acting on RNA 1, DEAH box helicase 9, and heterogeneous nuclear ribonucleoprotein L, suggesting that these RNA-binding proteins might be involved in the biogenesis of circRNAs during terminal B-cell differentiation. Notably, the circRNAs in human plasma cells were mainly derived from immunoglobulin (Ig) genes and represented the combinatorial clonal state of Ig loci.

Calcitonin gene-related peptide (CGRP) could induce the expression of IL-6 in macrophages^43^. In this process, mmu_circRNA_007893 mediated the IL-6 expression by working as an endogenous mmu-miR-485-5p sponge. When macrophages were stimulated by CGRP, mmu_circRNA_007893 was significantly increased. However, after silencing mmu_circRNA_007893, the level of mmu-miR-485-5p was increased while IL‑6 mRNA expression was decreased. During the process of immunosenescence, there was a significant feature that the proportion of CD8 T lymphocytes lacking CD28 expression would be increased^44^. Wang et al.^45^ discovered that circRNA_100783 in ageing human CD8^+^ T cells might function as a new biomarker for CD28-related CD8^+^ T-cell ageing. By further investigating the circRNA_100783-targeted miRNA-mRNA network, they observed that circRNA_100783 might be mainly related to alternative splicing events, the production of splice variants and the expression of phosphoprotein. Interestingly, circANRIL has been shown to disrupt pre-rRNA processing and ribosome biogenesis by binding to pescadillo homolog 1 in vascular smooth muscle cells and macrophages, leading to the nucleolar stress and activation of p53, which in turn induced apoptosis and inhibited proliferation^46^.

By measuring the expression profiles of circRNAs in 20 human tissues that were highly correlated with diseases, Maass et al.^47^ demonstrated that many circRNAs showed tissue-specific expression and could be closely related to the clinical phenotypes and mechanisms of human diseases. At the same time, they found that immune-related components, toll-like receptor 6 (TLR6), and myosin 1 F (MYO1F), could produce circRNAs in neutrophils, suggesting that the circRNAs were likely to be involved in neutrophil immune responses. In addition, Li et al.^48^ found that a W chromosome-linked circRNA was female-biased expression in a kind of flatfish (half-smooth tongue sole) and tended to be expressed in some immune tissues, especially head kidney and spleen. Importantly, the expression of this circRNA in spleen was significantly upregulated after infection, indicating that it might be related to the immune response. In summary, circRNAs actively participate in various biological processes in immune cells, such as differentiation, polarization, immune response, senescence, and apoptosis (Table 1).

**Table 1:** Association of circRNAs with the development and functions of immune cells

CircRNAs	Immune cells	Expression state	Functions	References
circ-FNDC3B, circ-ELK4, circ-MYBL1 and circ-SLFN12L	Differentiated lymphoid and myeloid cells	Differentially expressed in differentiated lymphoid and myeloid cells	Probably regulated the differentiation and cellular function in hematopoietic cells	^39^
189 differentially expressed circRNAs such as circRNA-010231	Macrophages	Differentially expressed in the M1 compared with the M2 macrophages	Involved in the differentiation and polarization of macrophages	^41^
1356 new identified circRNAs	Human humoral immune B cells	Differentially expressed in different plasma cells	Exquisitely controlled rearrangement of the Ig during the humoral immune response	^42^
mmu_circRNA_007893	Macrophages	Upregulated in CGRP-stimulated macrophages	Mediated the IL-6 expression by working as an mmu-miR-485-5p sponge	^43^
circRNA_100783	CD8^+^ T cells	Upregulated in C1 (CD28(+)CD8^+^ vs CD28(−)CD8^+^ T cells in the elderly) and C4 (CD28(−)CD8^+^ T cells in the elderly vs in the adult) cross-comparisons	Possibly regulated phosphoprotein-related signal transduction on CD28-dependent CD8^+^ T-cell ageing	^45^
circANRIL	Macrophages	-	Disrupted pre-rRNA processing and ribosome biogenesis by binding to PES1, which induced apoptosis and inhibited proliferation	^46^
circRNAs from TLR6 and MYO1F	Neutrophils	-	Possibly affected the expression of parental immune genes	^47^

*Ig* Immunoglobulin, *CGRP* Calcitonin gene-related peptide, *IL-6* Interleukin-6, *PES1* Pescadillo homolog 1, *TLR6* Toll-like receptor 6, *MYO1F* Myosin 1F

### circRNAs in immune regulation

In recent years, increasing evidence linked circRNAs to immune regulation under multifarious physiological and pathological conditions, including anti-infection immunity^49,50^, tumor immunity^51,52^, the activation of inflammation^53^, and even organ transplantation^54^. Through next-generation sequencing technology, Ma et al.^55^ found that 123 circRNAs were differentially expressed in Mock- and transmissible gastroenteritis virus (TGEV)-infected porcine intestinal epithelial cell line. Furthermore, Kyoto Encyclopedia of Genes and Genomes (KEGG) analysis suggested that the mRNAs in circRNA-miRNA-mRNA regulatory network were most significantly involved in inflammation and immune response, including retinoic acid-inducible gene-I (RIG-I)-like receptor, tumor necrosis factor (TNF), NOD-like receptor (NLR), TLR, and nuclear factor-κB (NF-κB) pathway. Of note, ssc_circRNA_009380 could promote the activation of NF-κB pathway via interacting with miR-22, thereby mediating TGEV-induced inflammation. Analogously, another study analyzed the circRNA expression profiles and circRNA-associated competing endogenous RNA (ceRNA) network of early HIV infection (EHI) patients^56^. The results indicated that 1365 circRNAs were abnormally expressed in HARRT-naive EHI patients in contrast to healthy controls, and the targeting mRNAs among the ceRNA networks were mainly related to inflammatory response, immune response and defense response to virus infection. Actually, circRNAs were closely related to the immune factors NF90/NF110 in viral infection^29^. Specifically, NF90/NF110 enhanced pre-mRNA back-splicing by stabilizing the intron complementary sequence pairs in the nucleus and interacted with mature circRNAs to form complexes in the cytoplasm. After viral invasions, NF90/NF110 in the nucleus were transported to the cytoplasm, and then the levels of circRNAs were reduced. At the same time, NF90/NF110 could be released from the complexes and subsequently suppressed viral replication by binding to viral mRNAs.

Meaningfully, Fu et al.^57^ found that 171 circRNAs were dysregulated in peripheral blood mononuclear cells (PBMCs) of patients with active tuberculosis (TB). Of these, circRNA_103017, circRNA_101128, and circRNA_059914 were expected to serve as new biomarkers for active TB. What’s more, circRNA_101128 could contribute to the pathogenesis of TB by regulating miRNA let-7a. In LPS-stimulated mouse macrophages, Ng et al.^58^ observed that one circRNA, mcircRasGEF1B, was regulated by TLR4 pathway. Moreover, the knockdown of mcircRasGEF1B reduced the expression of mature intercellular adhesion molecule-1 (ICAM-1) via modulating the stability of ICAM-1 mRNAs. Noteworthily, ICAM-1 was related to the pulmonary neutrophil recruitment in LPS-induced airway disease^59^, and could also suppress the polarization of M2 macrophages through the blockade of efferocytosis in tumor microenvironment^60^, implicating its various roles in innate immune response.

**Fig. 2:**
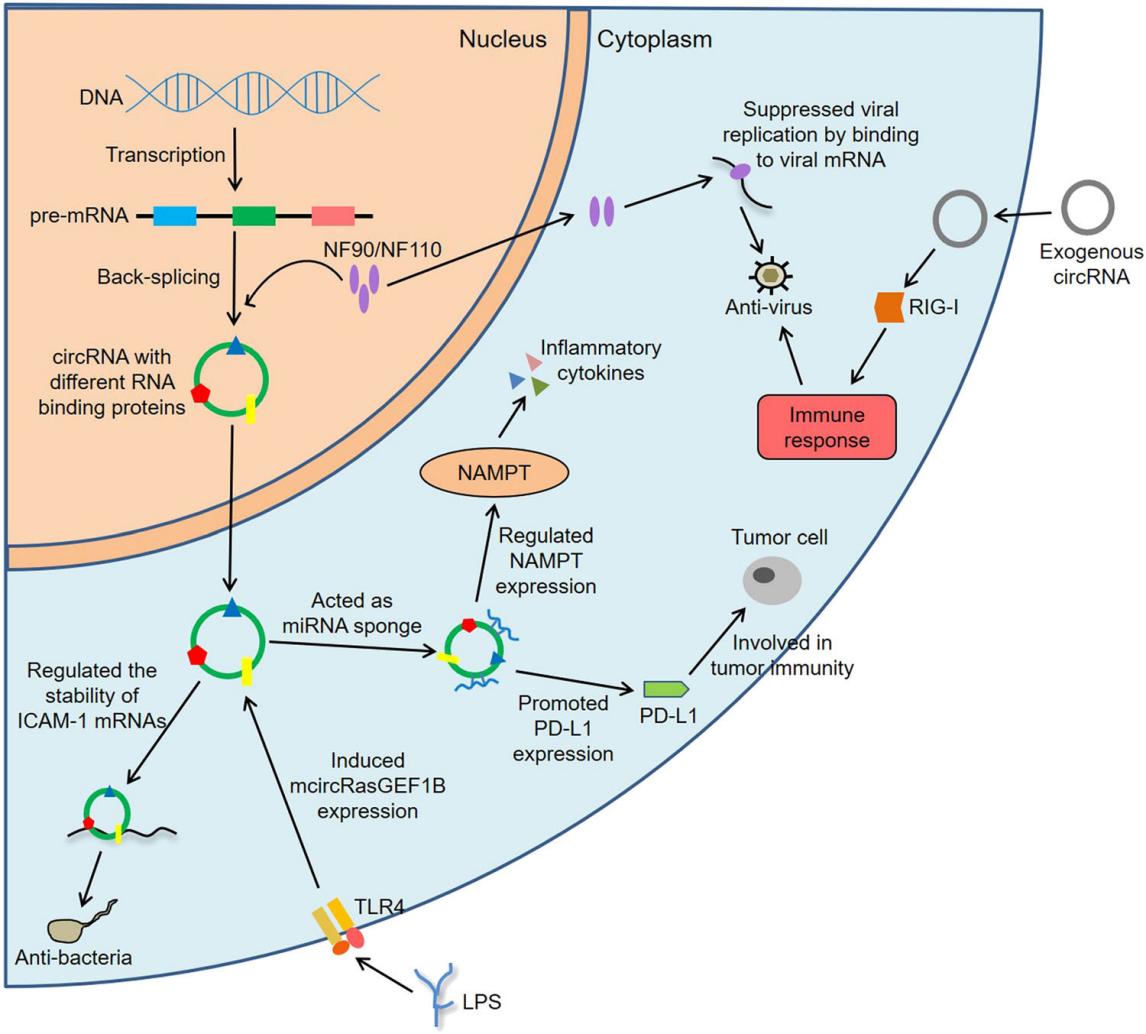
CircRNAs in immune regulation. NF90/NF110 enhance back-splicing by stabilizing the intron complementary sequence pairs in the nucleus and are exported to the cytoplasm to suppress viral replication after viral infection. The exogenous circRNA induces innate immune response by activating RIG-I, whereas the endogenous circRNA binds to different RNA-binding proteins that reflect its endogenous biogenesis. hsa_circ_0005105 facilitates the expression of inflammatory cytokines by regulating the miR-26a targeted NAMPT. has_circ_0020397 promotes the expression of PD-L1 by binding to miR-138, thereby participating in tumor immunity. In addition, mcircRasGEF1B induced by LPS is involved in anti-bacteria immunity by modulating the stability of ICAM-1 mRNAs

A recent study showed that hsa_circ_0005105 could facilitate the expression of inflammatory cytokines by regulating the miR-26a-targeted nicotinamide phosphoribosyltransferase, which provided a new target for the treatment of osteoarthritis (OA)^61^. In contrast to non-lesional skin of severe acne patients, Liang et al.^62^ discovered that up to 538 circRNAs were aberrantly expressed in adjacent lesional skin, and these circRNAs were mainly connected with the biological pathways such as inflammation, metabolism, and immune response. In addition, has_circ_0020397 could promote the expression of telomerase reverse transcriptase and programmed death-ligand 1 (PD-L1) by binding to miR-138, thereby regulating the viability, apoptosis and invasion of colorectal cancer cells^63^. Studies have shown that PD-L1 is closely related to tumor escape from immune control^64,65^, so has_circ_0020397 may promote tumor development by regulating tumor immunity. These findings demonstrate that circRNAs are crucial participants in immune regulation (Fig. 2). Hence, it is foreseeable that dysregulation of these functions is very likely to be involved in the development of autoimmune diseases.

## circRNAs in autoimmune diseases

**Fig. 3:**
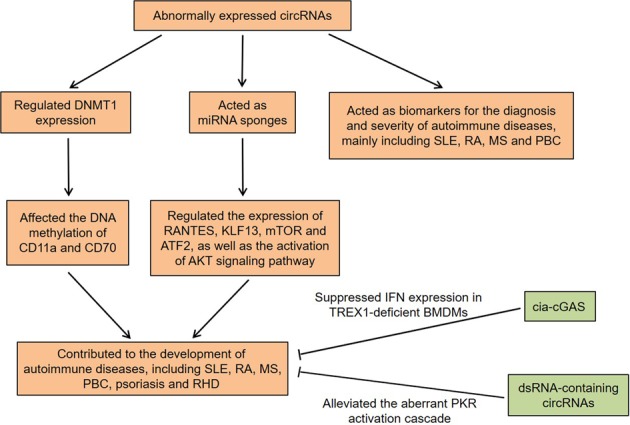
Roles of circRNAs in autoimmune diseases. CircRNAs contribute to the development of autoimmune diseases by regulating various biological processes, such as DNA methylation, immune response, and inflammatory response. Furthermore, circRNAs may be used as potential biomarkers for the diagnosis and severity of autoimmune diseases. The overexpression of cia-cGAS can suppress IFN expression in TREX1-deficient BMDMs. The overexpression of dsRNA-containing circRNAs alleviate the aberrant PKR activation cascade in SLE patient-derived cells

Autoimmune diseases, mainly characterized by a damaged immune system and the loss of immune tolerance to self-antigens, are a group of heterogeneous conditions^66^. Although the molecular mechanisms are still largely unknown, increasing evidence indicates that the complex interplay of environmental factors and epigenetic dysregulation facilitate the pathogenesis of these diseases in genetically susceptible individuals^67,68^. As described above, circRNAs are closely associated with the immune system. Meanwhile, recent studies have demonstrated that circRNAs are not only involved in the pathogenesis of autoimmune diseases, but also represent non-invasive biomarkers for them (Fig. 3).

### circRNAs in SLE

SLE is a chronic autoimmune disease that predominately affects women of childbearing age. Its main features are the autoreactive B and T lymphocytes as well as the overproduction of antibodies targeting self-antigens^69^. Unfortunately, SLE can result in multi-organ pathologies and a wide range of clinical manifestations, including arthritis, central nervous system disease, renal disease and skin disease^70^. Although SLE is immune-mediated, the pathogenic mechanisms are still not fully understood.

#### T-cell circRNAs in SLE

Recently, 127 differentially expressed circRNAs were identified in T cells of SLE patients, and a downregulated circRNA, hsa_circ_0045272, was further verified by quantitative PCR^71^. Mechanism research revealed that the knockdown of this circRNA significantly upregulated the early apoptosis and enhanced the production of IL-2 in activated Jurkat cells. Zhang et al.^72^ supported that hsa_circ_0012919 was aberrantly upregulated in CD4^+^ T cells of SLE patients. Meanwhile, the downregulation of hsa_circ_0012919 increased the expression of DNA methyltransferase 1 (DNMT1), whereas reduced the expression of CD70 and CD11a in CD4^+^ T cells from inactive and active SLE patients. The inhibition of hsa_circ_0012919 also rescued the DNA hypomethylation of CD70 and CD11a in CD4^+^ T cells of SLE patients, which could be reversed by downregulation of DNMT1. Strikingly, this circRNA could regulate the expression of regulated on activation, normal T cell expressed and secreted (RANTES) and Kruppel-like factor 13 (KLF13) by bonding to miR-125a-3p. It has been confirmed that migration rate of basophils to RANTES and monocyte chemotactic protein 1 (MCP-1) is remarkably increased in SLE patients, which is possibly associated with tissue damage in SLE^73^. KLF13 could positively regulate RANTES and was related to the expression of IL-4 in CD4^+^ T cells^74^.

#### PBMC circRNAs in SLE

Wang et al.^75^ found downregulation of circIBTK and upregulation of miR-29b in PBMCs of SLE patients, both of which were correlated with anti-double-stranded DNA, SLE Disease Activity Index (SLEDAI) score and complement component 3 (C3) level. Importantly, circIBTK could inhibit the DNA demethylation and activation of protein kinase B (AKT) by binding to miR-29b in SLE. Many studies have shown that AKT signaling pathway can regulate the functions of immune cells, and its dysregulation leads to the progression of SLE. For instance, AKT could coordinate IL-2 signaling and T-cell antigen receptor to hold the expression of adhesion molecules, cytolytic effector molecules, as well as cytokine and chemokine receptors in cytotoxic T cells^76^. Another research showed that hsa_circ_0049224 and has_circ_0049220 were underexpressed in PBMCs of inactive and active SLE patients^77^. Moreover, the levels of these two circRNAs were negatively correlated with SLEDAI and the degree of SLE severity, which indicated that they might be regarded as markers for the activity and severity of SLE.

#### Plasma circRNAs in SLE

In addition, hsa_circ_400011, hsa_circ_102584, hsa_circ_101471, and hsa_circ_100226 were abnormally expressed in plasma of SLE patients^9^. Through bioinformatics analysis, the researchers discovered multiple MREs of hsa_circ_100226, including hsa-miR-24-3p, hsa-miR-875-3p, hsa-miR-138-5p, hsa-miR-620, and hsa-miR-145-3p. Among them, decreased miR-138 could enhance NF-κB activation via suppressing the expression of p65 in the chondrocytes, triggering the inflammatory response^78^. Moreover, miR-138-5p regulated extracellular matrix catabolism and inflammation, thereby affecting the progression of OA^79^. Interestingly, the upregulated circRNA_002453 level in plasma of lupus nephritis (LN) patients was related to the severity of renal involvement^80^. Although the level of circRNA_002453 had no significant correlation with disease activity, it was positively associated with renal SLEDAI score and 24-hour proteinuria.

### circRNAs in RA

RA is a prevalent autoimmune disorder characterized by generalized inflammation in multiple joints, which always results in serious cartilage and bone erosion as well as articular deformation^81^. Rheumatoid factor (RF), anti-carbamylated protein (anti-CarP), and anti-cyclic citrullinated peptide-2 (anti-CCP2) are the most well-known autoantibodies in this disease^82^.

#### PBMC circRNAs in RA

Latest studies revealed that the levels of hsa_circ_0058794 and hsa_circ_0092285 were markedly increased in PBMCs of patients with RA, and the levels of hsa_circ_0038644 and hsa_circ_0088088 were decreased^83^. Actually, hsa_circ_0038644 was spliced from the protein kinase C beta gene, which was related to the activation of NF-κB^84^. Furthermore, the expression of ciRS-7 was significantly upregulated in RA patients, and it could reduce the inhibitory effect of miR-7 on mammalian target of rapamycin (mTOR) by inhibiting the function of miR-7^85^. As the phosphatidylinositol-3-kinase/AKT/mTOR (PI3K/AKT/mTOR) signaling pathway played an important role in synoviocyte proliferation and inflammatory responses^86,87^, ciRS-7 might be involved in the development of RA by regulating mTOR.

#### Other cell type circRNAs in RA

hsa_circ_0001859 was one of the differentially expressed circRNAs in synovial tissues of RA patients^88^. Mechanism studies found that this circRNA could promote activating transcription factors 2 expression and increase inflammatory activity by targeting miR-204/211. Furthermore, nuclear factor E2-related factor 2 (Nrf2), a potential therapeutic target for rheumatic diseases, could regulate many biological processes such as inflammation, immune response and cartilage and bone metabolism in the body^89^. By analyzing the circRNA expression profiles in the substantia nigra and corpus striatum of Nrf2-knockout mice, Yang et al.^90^ found that mmu_circRNA_34132, mmu-circRNA-015216 and mmu_circRNA_017077 were involved in the Nrf2-mediated neuroprotection against oxidative stress. Notably, the authors also uncovered that four mRNAs, Atp6v0a1, Atp6v0b, Atp6v0c, and Atp6v0e2, were enriched in RA pathway in the circRNA-miRNA-mRNA interaction network. Atp6v0c and Atp6v0e2 were potentially regulated by mmu_circRNA_017077 via binding to mmu-miR-346-3p, and Atp6v0e2 and Atp6v0a1 were potentially regulated by mmu_circRNA_34132 via binding to mmu-miR-346-3p as well. These results supported that mmu_circRNA_34132 and mmu_circRNA_017077 might participate in the Nrf2-mediated development of RA by serving as molecular sponges for mmu-miR-346-3p.

### circRNAs in MS

MS is a chronic disease of the central nervous system (CNS), and diffuse immune mechanisms as well as neurodegeneration are the underlying pathological processes in this disease. The peripheral immune response targeting the CNS occurs mainly in the early stage of MS, whereas immune process within the CNS dominates the progressive stage^91^. Most patients will develop permanent disability during the course of their disease, creating a huge burden for individual, family and society levels^92^.

Through the further characterization of Gasdermin B alternative splicing and back-splicing profiles, Cardamone et al.^93^ found that alternative splicing isoforms and an identified ecircRNA, containing exons 4 and 5, were significantly dysregulated in PBMCs of relapsing-remitting MS patients, which suggested that the abnormal RNA metabolism was involved in the pathogenesis of this disease. Metastasis associated lung adenocarcinoma transcript 1 (MALAT1) was an long non-coding RNA (lncRNA) that could regulate alternative splicing and has been shown to be associated with MS^94^. A systematic study found that the level of MALAT1 was upregulated in MS patients^95^. Meanwhile, 1114 alternative splicing events were significantly modulated and 49 circRNAs were differentially expressed in MALAT1-knockdown Jurkat T cells, a relevant cellular model for MS. Besides, the RNA-binding protein motif analysis showed a particular enrichment for the QKI in the exons modulated by MALAT1. Remarkably, QKI has been reported to regulate the formation of circRNAs^30^. These data indicate that MALAT1 dysregulation may lead to the development of MS by affecting splicing and back-splicing events.

### circRNAs in other autoimmune diseases

Psoriasis is an inflammatory disease that mainly affects the skin and joints, and its pathophysiological characteristics are abnormal proliferation of keratinocytes and infiltration of immune cells in the dermis and epidermis^96^. Recently, Liu et al.^97^ discovered six downregulated and 123 upregulated circRNAs in skin mesenchymal stem cells (S-MSCs) of psoriatic lesions. Pathway analysis observed that the significantly downregulated mRNAs in the lesions mainly enriched in Janus kinase-signal transducer and activator of transcription (JAK-STAT) signaling, which was reported to participate in immune regulation^98^. Of these, a circRNA chr2:206992521|206994966 could affect the activity of T lymphocytes in local lesions by regulating the secretion of certain cytokines, including IL-6, IL-11, and hepatocyte growth factor^97^. In addition, hsa_circ_0061012, hsa_circ_0003689, chr4:121675708|121732604, and hsa_circ_0003718 were abnormally expressed in psoriatic lesions and might promote disease progression by interacting with miRNAs associated with psoriasis^99,100^.

PBC is a cholestatic, autoimmune-mediated liver disease that slowly progresses to portal fibrosis and biliary cirrhosis^101^. By carrying out the circRNA expression profiles, Zheng et al.^102^ found 22 aberrantly expressed circRNAs in plasma of PBC patients. It was worth noting that the level of hsa_circ_402458 was higher in PBC patients not treated with ursodeoxycholic acid (UDCA) than in those treated with UDCA. At the same time, the authors showed that hsa_circ_402458 might target two miRNAs, hsa-miR-943, and hsa-miR-522-3p. For miR-522-3p, it might be an effective target for regulating chronic inflammatory disorder^103^. Therefore, it can be speculated that hsa_circ_402458 may function as a miRNA sponge to regulate inflammation-related signaling pathways, thus contributing to the development of PBC.

In addition, by studying the circRNA expression profiles in atrial tissues from patients with persistent atrial fibrillation (AF) with rheumatic heart disease, Hu et al.^104^ predicted the potential roles of the differentially expressed circRNAs. The results suggested that 51 circRNAs were upregulated, and 57 circRNAs were downregulated in AF tissues compared with controls, respectively. Gene Ontology (GO) analysis revealed that the most significantly enriched biological process term was muscle contraction, the most significantly enriched cellular component term was muscle myosin complex, and the most significantly enriched molecular function term was muscle alpha-actinin binding. Meanwhile, KEGG pathway analysis indicated that the main involved pathways were dilated cardiomyopathy and hypertrophic cardiomyopathy.

### circRNAs as potential biomarkers in autoimmune diseases

Owing to their stability, abundance, and evolutionary conservation, as well as their differential expression in patients with autoimmune diseases, circRNAs are likely to be potential biomarkers for these diseases. circPTPN22 derived from protein tyrosine phosphatase nonreceptor type 22 (PTPN22) was downregulated in the PBMCs of patients with SLE^105^. Importantly, the receiver operating characteristic (ROC) curve analysis showed that circPTPN22 had good diagnostic value for SLE. The downregulation of circPTPN22 was strongly negatively correlated with the SLEDAI scores, suggesting that this circRNA might be a biomarker for SLE diagnosis and disease severity. Zhang et al.^106^ observed that hsa_circRNA_407176 and hsa_circRNA_001308 were downregulated in both PBMCs and plasma of patients with SLE. Also, these two circRNAs in plasma and PBMCs might be candidate biomarkers for SLE, and their combination could improve the diagnostic efficiency. Even more, the level of hsa_circRNA_001308 was associated with C reactive protein and anti-sjögren’s syndrome-related antigen A in plasma, as well as leukopenia in PBMCs. Analogously, hsa_circ_0003090 and hsa_circ_0057762 in whole blood could differentiate the patients with SLE from the healthy controls, indicating that these two circRNAs might have potential value for SLE diagnosis^107^.

By the analysis of ROC curve, Ouyang et al.^108^ found that circRNA_104871 in PBMCs was a strong predictor for RA. Likewise, another study found that hsa_circ_0044235 was significantly decreased in peripheral blood of patients with RA^109^. Meaningfully, according to the risk score based on hsa_circ_0044235, the researchers could effectively distinguish the patients with RA from those with SLE. Iparraguirre et al.^110^ indicated that circ_0035560 and circ_0005402 were underexpressed in peripheral blood leukocytes of MS patients and might function as dependable biomarkers for this disease. Interestingly, these two circRNAs were derived from annexin A2 (ANXA2), whose linear form was also downregulated in MS patients. Increasing evidence has shown that ANXA2 is involved in many autoimmune diseases, including antiphospholipid syndrome and LN, suggesting that circ_0035560 and circ_0005402 may be associated with the development of MS^111,112^. In summary, these studies provide a theoretical basis for the clinical application of circRNAs in autoimmune diseases.

## Conclusion and future perspectives

Indeed, increasing evidence has identified that circRNAs are active participants in multiple stages of immune-cell development and immune regulation. Furthermore, circRNAs may not only be diagnostic biomarkers for human autoimmune diseases, but also represent the disease activity or severity. More importantly, circRNAs contribute to the development of autoimmune diseases by acting as miRNA sponges to regulate many biological processes, including DNA methylation, immune response, and inflammatory response (Table 2). Therefore, elucidating the roles of circRNAs in the setting of autoimmune disease will be a promising field.

**Table 2:** Summary of circRNAs involved in autoimmune diseases

CircRNAs	Disease or model	Cell or tissue type	Functions	References
hsa_circ_0045272	SLE	T cells	Upregulated the early apoptosis of Jurkat cells and enhanced the production of IL-2 in activated Jurkat cells	^71^
hsa_circ_0012919	SLE	CD4^+^ T cells	Increased DNMT1 expression, reduced CD70 and CD11a expression, rescued the DNA hypomethylation of CD11a and CD70 in CD4^+^ T cells of SLE patients, as well as regulated the expression of RANTES and KLF13 by bonding to miR-125a-3p	^72^
circIBTK	SLE	PBMCs	Inhibited DNA demethylation and activation of AKT signaling pathway by binding to miR-29b	^75^
hsa_circ_0049224 and has_circ_0049220	SLE	PBMCs	Negatively correlated with SLEDAI and the degree of SLE severity	^77^
circPTPN22	SLE	PBMCs	Served as a biomarker for the diagnosis and severity of SLE	^105^
hsa_circ_400011, hsa_circ_102584, hsa_circ_101471, and hsa_circ_100226	SLE	Plasma	Possibly involved in the development of SLE by acting as miRNA sponges	^9^
circRNA_002453	SLE	Plasma	Associated with the renal SLEDAI score and 24-hour proteinuria	^80^
hsa_circRNA_407176 and hsa_circRNA_001308	SLE	Plasma and PBMCs	Served as biomarkers for SLE, and hsa_circRNA_001308 was correlated with CRP and anti-SSA in plasma, as well as leukopenia in PBMCs	^106^
hsa_circ_0003090 and hsa_circ_0057762	SLE	Whole blood	Served as biomarkers for the diagnosis of SLE, and hsa_circ_0057762 was positively associated with the SLEDAI-2K score	^107^
ciRS-7	RA	PBMCs	Reduced the inhibitory effect of miR-7 on mTOR by inhibiting the function of miR-7	^85^
circRNA_104871	RA	PBMCs	Served as a strong predictor for RA	^108^
hsa_circ_0001859	RA	Synovial tissues	Promoted ATF2 expression and increased inflammatory activity by targeting miR-204/211	^88^
mmu_circRNA_017077 and mmu_circRNA_34132	RA	Nrf2-knock-out substantia nigra and corpus striatum	Involved in the Nrf2-mediated development of RA by serving as sponges for mmu-miR-346-3p	^90^
hsa_circ_0044235	RA	Peripheral blood	Served as a biomarker for RA and effectively distinguished the patients with RA from those with SLE	^109^
An ecircRNA from GSDMB	MS	PBMCs	Involved in the pathogenesis of MS	^93^
49 differentially expressed circRNAs	MS	MALAT1-knockdown Jurkat T cells	Possibly led to the development of MS by affecting splicing and back-splicing events	^95^
circ_0005402 and circ_0035560	MS	Peripheral blood leukocytes	Derived from ANXA2 and served as potential biomarkers for MS	^110^
A circRNA chr2:206992521|206994966	Psoriasis	S-MSCs	Affected the activity of T lymphocytes by regulating the secretion of certain cytokines, including IL-6, IL-11, and hepatocyte growth factor	^97^
hsa_circ_0061012, hsa_circ_0003689, chr4:121675708|121732604 and hsa_circ_0003718	Psoriasis	Psoriatic lesions	Possibly promoted disease progression by interacting with miRNAs associated with psoriasis	^99, 100^
hsa_circ_402458	PBC	Plasma	Possibly acted as a miRNA sponge to moderate inflammation-related signaling pathways	^102^
108 differentially expressed circRNAs	RHD	Atrial tissues	Associated with the development of AF with RHD	^104^
cia-cGAS	An autoimmune disease model	TREX1-deficient BMDMs	Suppressed IFN expression in TREX1-deficient BMDMs	^114^
dsRNA-containing circRNAs	SLE	PBMCs	Alleviated the aberrant PKR activation cascade in SLE patient-derived cells	^117^

*SLE* Systemic lupus erythematosus, *RA* Rheumatoid arthritis, *MS* Multiple sclerosis, *PBC* Primary biliary cholangitis, *AF* Atrial fibrillation, *RHD* Rheumatic heart disease, *PBMCs* Peripheral blood mononuclear cells, *Nrf2* Nuclear factor E2-related factor 2, *MALAT1* Metastasis associated lung adenocarcinoma transcript 1, *S-MSCs* Skin mesenchymal stem cells, *TREX1* Three-prime repair exonuclease 1, *BMDMs* Bone marrow derived macrophages, *IFN* Interferon, *IL-2* Interleukin-2, *DNMT1* DNA methyltransferase 1, *AKT* Kinase B, *SLEDAI* SLE Disease Activity Index, *mTOR* Mammalian target of rapamycin, *CRP* C reactive protein, *anti-SSA* Anti-sjögren’s syndrome-related antigen A, *C3* Complement component 3, *ATF2* Activating transcription factors 2, *miRNA* MicroRNA, *GSDMB* Gasdermin B, *ANXA2* Annexin A2, *dsRNA* Double-stranded RNA, *PKR* Protein kinase

Recently, Chen et al.^113^ found that the purified exogenous circRNA could induce innate immune response and confer a protective effect on viral infection by activating RIG-I. Further exploration indicated that cells could distinguish between self-nonself circRNAs based on the introns that produced them, and the reason might be that mature human circRNAs always bound to different RNA-binding proteins that reflect their endogenous splicing and biogenesis. In this regard, we speculate that the abnormal circRNAs in vivo, like foreign circRNAs, are involved in autoimmune diseases by activating the immune signaling. Interestingly, Xia et al.^114^ demonstrated that a novel circRNA, cia-cGAS, could protect dormant long-term-hematopoietic stem cells from cyclic GMP-AMP synthase (cGAS)-mediated exhaustion by suppressing the enzymatic activity of cGAS under homeostatic conditions. Meanwhile, the binding affinity of cia-cGAS for cGAS was stronger than that of self-DNA, thereby inhibiting cGAS-mediated generation of type I IFNs to maintain dormant HSCs. Three-prime repair exonuclease 1 (TREX1) was a major 3ʹ → 5ʹ DNA exonuclease, whose dysregulation has been associated with some autoimmune diseases^115,116^. Conspicuously, the overexpression of cia-cGAS could suppress IFN expression in TREX1-deficient bone marrow derived macrophages (BMDMs), indicating that cia-cGAS could restrain autoimmune signaling in TREX1-deficient cells^114^. Thus, cia-cGAS might act as a potential target for the treatment of autoimmune diseases by antagonizing cGAS. Recently, Liu et al.^117^ found that endogenous circRNAs tended to form 16-26 bp intra-molecular RNA duplexes and inhibited double-stranded RNA (dsRNA)-activated protein kinase (PKR) activity by preferentially binding to PKR. The activity of RNase L in PBMCs derived from SLE patients was enhanced, accompanied by reduced circRNA expression and augmented PKR phosphorylation. Importantly, overexpression of dsRNA-containing circRNAs could strongly attenuate the aberrant PKR activation cascade in SLE patient-derived cells, suggesting that circRNAs might serve as potential targets for the treatment of autoimmune diseases.

However, several important questions merit further resolution. To date, we have detailed the close relationship between autoimmune diseases and circRNAs, but little is known about the molecular mechanisms that trigger the pathogenesis. CircRNAs that act as endogenous miRNA sponges have been studied widely, but few circRNA/miRNA interactions have been experimentally validated in immunity^61,63,118^. It is suggested that exploring other mechanisms may improve the functional description of circRNAs in immunological contexts. Furthermore, circRNAs can exert potential biomarkers for various autoimmune diseases. Nonetheless, in many studies, the sample size of patients is relatively small, or the sample source has some limitations, which may affect the validity and universality of the conclusions that circRNAs can serve as biomarkers. Moreover, in-depth studies of circRNAs’ biogenesis, accumulation in the cytoplasm, and even post-transcriptional modifications may increase our understanding of their biological functions.

In conclusion, continued investigation into circRNAs may yield more discoveries in the pathogenesis of autoimmune diseases and broaden the spectra of diagnosis and therapy for these diseases in the future.
